# Facility Type Predicts Completeness of Oncologic Resection and Survival in Biliary Tract Cancers

**DOI:** 10.1007/s12029-026-01421-1

**Published:** 2026-02-19

**Authors:** Trisha Lal, Weichuan Dong, Sami O. Abul-Khoudoud, Amit Mahipal, John B. Ammori, Richard S. Hoehn

**Affiliations:** 1 Division of Surgical Oncology, University Hospitals, Cleveland Medical Center, 11100 Euclid Ave, Cleveland, OH 44106 USA; 2 School of Medicine, Case Western Reserve University, Cleveland, OH 44106 USA; 3 Department of Population and Quantitative Health Sciences, School of Medicine, Case Western Reserve University, Cleveland, OH 44106 USA; 4 Houston Methodist Research Institute, 77030 Houston, TX USA; 5 Department of Oncology, University Hospitals, Cleveland Medical Center, Cleveland, OH 44106 USA; 6 Case Comprehensive Cancer Center, Cleveland, OH 44106 USA

**Keywords:** Biliary tract cancers, Surgical outcomes, Lymphadenectomy, Surgical margins, Facility type

## Abstract

**Purpose:**

Complete Oncologic Resection (COR) for biliary tract cancers (BTC) includes negative margin status and adequate lymphadenectomy, defined as retrieval of ≥ 6 lymph nodes. Whether these standards and their association with survival vary across facility types and BTC subtypes remains unclear.

**Methods:**

Using the National Cancer Database (2004–2022), we identified adults undergoing curative-intent resection for Stage I-III BTC, excluding T1a gallbladder cancer. Facility types were compared for COR using nested logistic regression, adjusting for demographic and tumor factors. Overall survival (OS) was assessed using multivariable Cox proportional hazards models, with Kaplan-Meier (KM) analyses used to describe unadjusted, stratified survival patterns.

**Results:**

Among 13,250 adults, 51.6% were treated at academic centers. Negative margins were achieved in 79.5% of academic versus 79.0% elsewhere, while adequate lymphadenectomy occurred in 48.2% versus 38.2%, respectively (*p* < 0.001). Non-academic programs had lower odds of COR (OR 0.78–0.94), and gallbladder cancer had the lowest odds of each COR component relative to other BTC subtypes (negative margins: OR 0.80, 95% CI 0.69–0.93; adequate lymphadenectomy: OR 0.77, 95% CI 0.68–0.88). Cox estimates showed higher mortality with positive margins (HR 1.80, 95% CI 1.71–1.89), inadequate lymphadenectomy (HR 1.22, 95% CI 1.16–1.28), incomplete oncologic surgery (HR 1.36, 95% CI 1.30–1.43), and non-academic facilities (HR 1.17, 95% CI 1.12–1.23).

**Conclusion:**

Academic centers more frequently deliver COR and are associated with improved survival, but lymphadenectomy benchmarks remain unmet across facility types. System-level interventions, including multidisciplinary collaboration and referral networks, are necessary to expand access to high-quality care and improve outcomes.

**Supplementary Information:**

The online version contains supplementary material available at 10.1007/s12029-026-01421-1.

## Introduction

Biliary tract cancers (BTC), including intrahepatic cholangiocarcinoma, extrahepatic cholangiocarcinoma, and gallbladder cancer, are rare but expected to continue to rise in incidence [1, 2]. Unfortunately, estimated 5-year survival rates are as low as 20%, primarily due to late-stage detection and high recurrence rates [3]. As surgery offers the only potential cure [1, 3], the National Comprehensive Cancer Network (NCCN) designates two primary quality measures for BTC resection: complete microscopic margin negativity and adequate lymphadenectomy [4]. Additionally, while the NCCN recommends a site-appropriate regional lymphadenectomy, the American Joint Committee on Cancer (AJCC) 8th edition and most registry studies consider examination of six or more lymph nodes as the benchmark for complete staging [5].

Previous studies have evaluated BTC subtypes separately, consistently demonstrating that academic programs achieve superior surgical outcomes and survival [6–10]. A broader BTC analysis series has also revealed suboptimal adherence to guideline-recommended adjuvant chemotherapy at non-academic centers [11]. However, no study has assessed whether Commission on Cancer (CoC) facility types uniformly meet surgical benchmarks when BTCs are treated as a single cohort, nor have outcomes across subtypes been directly compared.

In this context, we performed a retrospective study of adults undergoing curative-intent resection for BTC in the National Cancer Database (NCDB) (2004–2022). We compared the odds of complete oncologic resection (COR), defined as concurrent negative margins and retrieval of 6 or more nodes, and its components, across CoC facility types. Within the same cohort, we stratified outcomes by BTC subtype to more precisely identify facility-type targets for system-level improvements. Finally, we assessed overall survival stratified by margin status or lymphadenectomy adequacy and facility type.

## Methods

### Data Source and Cohort Selection

This retrospective cohort study used data from the 2022 Participant User File (PUF) of the National Cancer Database (NCDB), which captures approximately 72% of all newly diagnosed cancers in the United States [12]. We identified adults (aged 18 years or older) diagnosed with intrahepatic cholangiocarcinoma (IHC), extrahepatic cholangiocarcinoma (EHC), or gallbladder cancer (GBC) between January 1, 2004, and December 31, 2022, who underwent definitive resection defined by NCDB surgery codes 20–80. These encompass hepatic and bile duct resections, pancreaticoduodenectomy, cholecystectomy with regional lymphadenectomy, and combined organ resections performed with curative intent. We restricted inclusion to patients with AJCC Stage I-III disease and excluded patients with T1a GBC, who typically undergo simple cholecystectomy.

We excluded cases coded as local tumor destruction, local tumor excision, or unknown surgery type, as well as those with missing key demographic or clinical data, assuming that missingness was completely at random. This study adhered to the Strengthening the Reporting of Observational Studies in Epidemiology (STROBE) reporting guideline [13]. It was exempt from our Institutional Review Board review because the NCDB is a de-identified database.

### Outcomes

Our primary outcome was COR, a composite measure defined as the presence of both a negative microscopic margin and adequate lymphadenectomy. Margin status was considered negative if all margins were grossly and microscopically tumor-free, or positive if any residual tumor (microscopic, macroscopic, or indeterminate) was present. Adequate lymphadenectomy was defined, according to AJCC guidelines, as the removal of 6 or more regional lymph nodes. Secondary outcomes included overall survival (OS), measured from the date of diagnosis to the date of death, with patients who were alive but lost to follow-up censored.

### Covariates

Extracted variables include patient demographics (age, sex, race/ethnicity), clinical factors (Charlson-Deyo Score (CDS), tumor site, AJCC pathologic stage), treatment details (insurance payer, travel distance, treatment at multiple facilities, adjuvant therapy receipt), surgical approach (open, minimally invasive, robotic), CoC facility type, and facility annual surgical volume. Facility types were defined according to accreditation and volume: academic/research programs (≥ 500 new cases/year with postgraduate training in ≥ 4 specialties), comprehensive community cancer programs (CCPs) (≥ 500 cases/year without postgraduate training), integrated network cancer programs (centralized governance of multisite cancer services), and CCPs (100–499 cases/year) [14]. For select analyses, these were dichotomized into academic (academic/research programs) vs. non-academic (all other facility types). Annualized surgical volume was calculated for each facility by dividing the number of BTC resections by the years the facility reported resections to the NCDB. A binary volume variable was created using the top quartile (≥ 8.7 resections/year) to define high-volume centers.

### Statistical Analysis

The overall goal of this study was to compare surgical quality across facility types. Descriptive statistics characterized patient, tumor, and treatment variables, stratified by academic and non-academic facilities. Continuous variables were summarized using medians and interquartile ranges (IQRs), and compared using the Kruskal-Wallis test unless normality was confirmed. Categorical variables were summarized as frequencies and percentages.

Associations between facility type and surgical quality measures were evaluated using nested logistic regression models, adapted from prior analyses [15, 16]. Model 1 was unadjusted and estimated the crude association between facility type and outcome. Model 2 was adjusted for demographic (age, sex) and clinical factors (CDS, primary site, AJCC pathologic stage, and surgical approach), which reflect baseline characteristics that influence both patient selection and surgical complexity. This model was also used for site-specific analyses to maintain power and avoid over-adjustment. Model 3 further adjusted for race and ethnicity to probe the role of structural racism, implicit bias, and differential trust in healthcare systems on access to high-quality measures.

Overall survival (OS) was first described using Kaplan-Meier (KM) methods and log-rank tests to provide unadjusted comparisons across margin status, lymphadenectomy adequacy, and oncologic resection. Curves were truncated when the number at risk fell below 10% of the original cohort to comply with the NCDB’s minimum cell-count policy. Pairwise log-rank tests with Holm multiplicity adjustment were conducted but interpreted descriptively given the absence of covariate adjustment.

To evaluate independent associations between surgical quality, facility type, and survival, we constructed multivariable Cox proportional hazards models including all covariates from Model 3 (age, sex, CDS, primary site, stage, surgical approach, and race and ethnicity), as well as receipt of adjuvant chemotherapy. Separate models examined margin status, lymphadenectomy, and oncologic resection. In response to reviewer feedback, we additionally tested an interaction between facility type and lymphadenectomy adequacy. The proportional-hazards assumption was assessed using Schoenfeld residuals. As a sensitivity analysis, patients who died within 90 days of surgery were censored at 3 months to account for perioperative mortality. OS analyses were limited to cases diagnosed through 2021, as the 2022 NCDB file does not include survival follow-up per NCDB standard reporting.

Statistical significance was defined as a two-sided *p*-value < 0.05. All analyses were performed using RStudio (version 2025.05.0 + 496).

## Results

A total of 13,250 patients met the inclusion criteria (Supplementary Fig. 1), of which 6,831 (51.6%) were treated at academic/research programs (Table 1). Of non-academic programs, 3,467 (26.2%), 2,502 (18.9%), and 450 (3.4%) patients received care at comprehensive CCPs, integrated network cancer programs, and CCPs, respectively. Over half of resections at academic sites occurred at high-volume centers (53.2%), compared with 15.0% at non-academic sites (*p* < 0.001). Patients treated at academic centers were younger (median age, 68 vs. 70 years), had a lower comorbidity burden (CDS 0: 67.7% vs. 63.6%), and traveled farther for care (median, 19.10 vs. 9.50 miles) than those at non-academic sites (all *p* < 0.001). Academic centers had a lower proportion of GBC (31.2% vs. 54.8%) and a higher proportion of IHC (22.7% vs. 12.6%) and EHC (46.1% vs. 32.6%) compared with non-academic facilities (all *p* < 0.001). Stage at diagnosis also varied across subtypes (Supplementary Fig. 2): GBC were largely Stage III (52%), IHC Stage I (42%), and EHC Stage II (60%).

Patients who underwent surgery at academic centers also received COR more frequently (39.7% vs. 31.5%), had higher rates of adequate lymphadenectomy (48.2% vs. 38.2%), and slightly more frequent margin-negative resection (79.5% vs. 79.0%) (all *p* < 0.001). Receipt of adjuvant therapy also differed significantly across facility types: academic centers had higher rates of chemotherapy (84.2% vs. 68.7%) but slightly lower rates of radiation therapy (24.8% vs. 25.3%) (both *p* < 0.001). Of note, to contextualize the low adequacy rates for lymphadenectomy, we confirmed that all patients across facility types had at least one lymph node examined.

**Table 1:** Patient characteristics by facility type

*n*		Academic	Non-academic	*p*
		6831 (51.6)	6419 (49.4%)	
Age (median [IQR])		68.00 [60.00, 75.00]	70.00 [62.00, 77.00]	**< 0.001**
Sex (%)	**Male**	3490 (51.1)	2685 (41.8)	**< 0.001**
	**Female**	3341 (48.9)	3734 (58.2)	
Race/Ethnicity (%)	**Non-Hispanic White**	4635 (67.9)	4460 (69.5)	**< 0.05**
	**Non-Hispanic Black**	769 (11.3)	703 (11.0)	
	**Hispanic**	732 (10.7)	641 (10.0)	
	**Asian**	468 (6.9)	361 (5.6)	
	**Other**	227 (3.3)	254 (4.0)	
Insurance (%)	**Private**	2266 (33.2)	1904 (29.7)	**< 0.001**
	**Medicare**	3672 (53.8)	3871 (60.3)	
	**Medicaid**	526 (7.7)	370 (5.8)	
	**Not insured**	186 (2.7)	144 (2.2)	
	**Other/Unknown**	181 (2.6)	130 (2.0)	
Charlson Deyo Score (%)	**0**	4625 (67.7)	4080 (63.6)	**< 0.001**
	**1**	1472 (21.5)	1494 (23.3)	
	**2**	414 (6.1)	486 (7.6)	
	**3**	320 (4.7)	359 (5.6)	
Primary Site (%)	**Gallbladder**	2132 (31.2)	3518 (54.8)	
	**Intrahepatic bile duct**	1551 (22.7)	807 (12.6)	**< 0.001**
	**Extrahepatic bile duct**	3148 (46.1)	2094 (32.6)	
Stage (%)	**I**	1386 (20.3)	1131 (17.6)	
	**II**	3137 (45.9)	2744 (42.7)	
	**III**	2308 (33.8)	2544 (39.6)	
Treatment at Multiple Facilities (%)		1373 (20.1)	1451 (22.6)	**< 0.001**
Distance in Miles (median[IQR])		19.10 [7.10, 54.25]	9.50 [4.40, 23.20]	**< 0.001**
Annual Surgical Volume (%)	**High**	3617 (52.9)	964 (15.0)	**< 0.001**
	**Low**	3214 (47.1)	5455 (85.0)	
Region (%)	**Northeast**	2107 (30.8)	1021 (15.9)	**< 0.001**
	**Midwest**	1296 (19.0)	1404 (21.9)	
	**South**	2451 (35.9)	2599 (40.5)	
	**West**	977 (14.3)	1395 (21.7)	**< 0.001**
Adequate Margins (%)		5431 (79.5)	5070 (79.0)	**< 0.001**
Adequate Lymphadenectomy (%)		3292 (48.2)	2450 (38.2)	**< 0.001**
Complete Oncologic Resection (%)		2715 (39.7)	2021 (31.5)	**< 0.001**
Adjuvant Therapy Receipt (%)	**Chemotherapy**	5754 (84.2)	4413 (68.7)	**< 0.001**
	**Radiation**	1696 (24.8)	1623 (25.3)	**< 0.001**
Surgical Approach (%)	**Open**	5151 (75.4)	3818 (59.5)	**< 0.001**
	**Minimally invasive**	1393 (20.4)	2232 (34.8)	
	**Robotic**	287 (4.2)	369 (5.7)	

### Complete Oncologic Resection

**Fig. 1:**
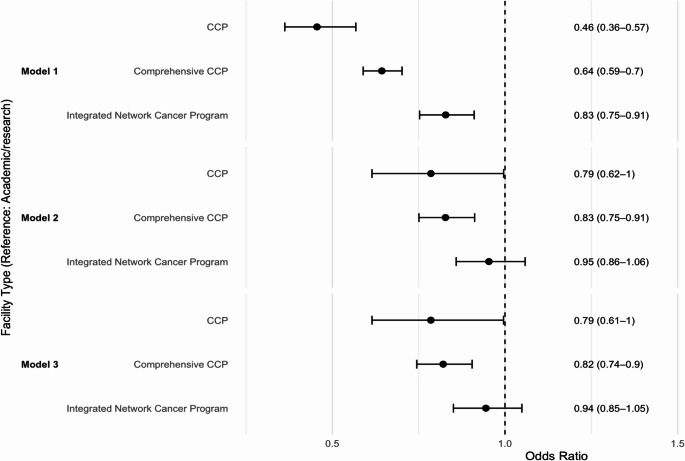
Odds of complete oncologic resection by facility type. Forest plots of odds ratios (95% CI) for complete oncologic resection at community cancer programs (CCP), comprehensive CCPs, and integrated network cancer programs versus academic/research centers (reference, OR = 1) across three logistic regression models

In the fully adjusted model (Model 3), only comprehensive CCPs had lower odds of achieve COR compared with academic centers (OR 0.82, 95% CI 0.74–0.90), while the estimates for CCPs (OR 0.79, 95% CI 0.61-1.00) and integrated network programs (OR 0.94, 95% CI 0.85–1.05) did not reach statistical significance (Fig. 1). Among patient-level factors, both non-Hispanic Black (OR 0.81, 95% CI 0.71–0.93) and Hispanic patients (OR 0.82, 95% CI 0.72–0.94) had lower odds of undergoing optimal resection than non-Hispanic White patients. Compared with GBC, patients with EHC had markedly higher odds of achieving COR (OR 5.03, 95% CI 4.56–5.56), whereas IHC did not differ significantly (OR 0.98, 95% CI 0.86–1.12). Minimally invasive surgery was associated with slightly lower odds of COR compared with open surgery (OR 0.84, 95% CI 0.76–0.93), while robotic surgery showed no significant difference.

### Resection Margins

**Fig. 2:**
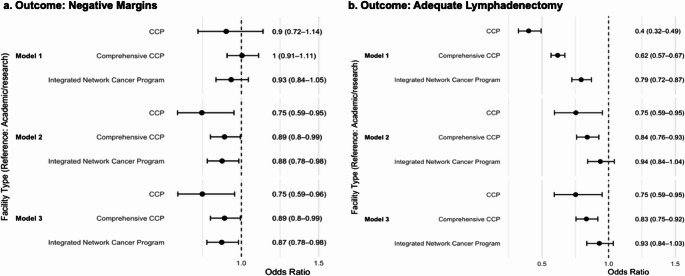
Odds of negative margins and adequate lymphadenectomy by facility type. Forest plots of odds ratios (95% CI) for (**a**) negative resection margins and (**b**) adequate lymphadenectomy at community cancer programs (CCP), comprehensive CCPs, and integrated network cancer programs versus academic/research centers (reference, OR = 1) across three logistic regression models

**Fig. 3:**
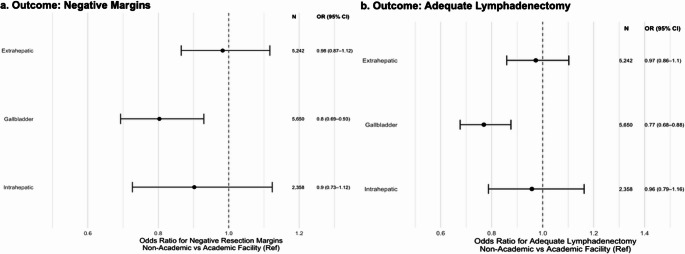
Odds of negative margins and adequate lymphadenectomy by primary tumor site. Forest plots of adjusted odds ratios (95% CI) comparing non-academic versus academic centers (reference, OR = 1) for (**a**) negative resection margins and (**b**) adequate lymphadenectomy across extrahepatic, gallbladder, and intrahepatic biliary cancers. Each point is labeled with the number of cases (N) and the OR (95% CI)

After adjusting for all covariates, odds of negative resection margins were significantly lower at CCPs (OR 0.75, 95% CI 0.59–0.96), comprehensive CCP (OR 0.89, 95% CI 0.80–0.99), and integrated network programs (OR 0.87, 95% CI 0.78–0.98) compared with academic centers (Fig. 2a). Stratified by tumor site, academic centers similarly outperformed others in the resection of GBC (OR 0.80, 95% CI 0.69–0.93), while differences of IHC (OR 0.90, 95% CI 0.73–1.12) and EHC (OR 0.98, 95% CI 0.87–1.12) were not statistically significant (Fig. 3a).

## Adequate Lymphadenectomy

Similarly, non-academic centers were less likely to achieve adequate lymphadenectomy than academic/research programs: CCPs had an OR of 0.40 (95% CI 0.75–0.95), and comprehensive CCPs had an OR of 0.83 (95% CI 0.75–0.92) (Fig. 2b). Subtype analysis revealed a persistent disparity in lymphadenectomy quality for GBC (OR 0.77, 95% CI 0.68–0.88), but no difference for IHC (OR 0.96, 95% CI 0.79–1.16) or EHC (OR 0.97, 95% CI 0.86–1.10) between non-academic and academic centers (Fig. 3b).

### Survival Outcomes

In the overall cohort, the three- and five-year overall survival estimates were 50.9% (95% CI, 50.0-51.8%) and 38.3% (95% CI, 37.4–39.3%), respectively. As KM curves do not adjust for differences in disease severity or patient mix, they are presented as descriptive summaries. When stratified by margin status and facility type, KM curves showed that negative margins at academic centers were associated with the most favorable survival, whereas positive margins at non-academic centers were associated with the poorest outcomes (Supplementary Fig. 3a). These differences were statistically significant on pairwise Holm-adjusted log-rank testing (all *p* < 0.001). In adjusted Cox models, the same pattern remained, with positive margins associated with almost double the risk of death compared with negative margins (HR 1.77, 95% CI 1.66–1.89), and treatment at non-academic centers remained associated with a 12% increase in mortality (HR 1.12, 95% CI 1.06–1.19) (Table 2).

**Table 2:** Adjusted Cox proportional hazards models for overall survival including margin Status, Lymphadenectomy, or oncologic resection

		Margin Status	Lymphadenectomy	Oncologic Resection
		HR (95% CI)	HR (95%CI)	HR (95%CI)
Age		1.01 (1.01–1.02)	1.01 (1.01–1.02)	1.01 (1.01–1.02)
Sex	**Female**	ref	ref	ref
	**Male**	1.08 (1.02–1.15)	1.08 (1.02–1.15)	1.08 (1.02–1.15)
Race/Ethnicity	**Non-Hispanic White**	ref	ref	ref
	**Non-Hispanic Black**	1.07 (0.97–1.18)	1.06 (0.97–1.17)	1.05 (0.96–1.16)
	**Hispanic**	0.82 (0.74–0.92)	0.83 (0.74–0.92)	0.82 (0.74–0.92)
	**Asian**	0.79 (0.69–0.90)	0.79 (0.69–0.90)	0.78 (0.68–0.89)
	**Other**	0.93 (0.79–1.09)	0.93 (0.79–1.10)	0.94 (0.80–1.10)
Charlson Deyo Score	**0**	ref	ref	ref
	**1**	1.07 (1.00-1.15)	1.09 (1.01–1.17)	1.09 (1.02–1.17)
	**2**	1.11 (0.99–1.26)	1.15 (1.02–1.30)	1.15 (1.02–1.30)
	**3**	1.32 (1.15–1.52)	1.37 (1.19–1.58)	1.32 (1.18–1.55)
Primary Site	**Gallbladder**	ref	ref	ref
	**Intrahepatic bile duct**	0.96 (0.87–1.06)	1.03 (0.93–1.14)	1.01 (0.92–1.12)
	**Extrahepatic bile duct**	1.26 (1.17–1.36)	1.52 (1.40–1.65)	1.54 (1.42–1.66)
Stage	**I**	ref	ref	ref
	**II**	1.46 (1.31–1.63)	1.50 (1.34–1.68)	1.51 (1.35–1.69)
	**III**	2.25 (2.01–2.52)	2.48 (2.22–2.78)	2.45 (2.19–2.74)
Facility Type	**Academic**	ref	ref	ref
	**Non-Academic**	1.12 (1.06–1.19)	1.12 (1.05–1.19)	1.12 (1.06–1.19)
Margin Status	**Negative**	ref	-	-
	**Positive**	1.77 (1.66–1.89)	-	-
Lymphadenectomy	**Adequate (ref)**	-	ref	-
	**Inadequate**	-	1.23 (1.15–1.31)	-
Oncologic Resection	**Complete (ref)**	-	-	ref
	**Incomplete**	-	-	1.38 (1.30–1.48)
Surgical Approach	**Open**	ref	ref	ref
	**Minimally invasive**	0.83 (0.77–0.90)	0.83 (0.77–0.89)	0.82 (0.76–0.89)
	**Robotic**	0.77 (0.66–0.89)	0.77 (0.66–0.90)	0.77 (0.66–0.89)
Chemotherapy Receipt	**Received (ref)**	ref	ref	ref
	**Refused/Contraindicated**	1.52 (1.39–1.66)	1.50 (1.37–1.64)	1.50 (1.37–1.63)
	**Not received**	1.40 (1.04–1.90)	1.35 (1.00-1.83)	1.35 (1.00-1.82)

When stratified by lymphadenectomy adequacy and facility type, unadjusted survival curves demonstrated only modest separation (Supplementary Fig. 3b). The poorest survival occurred among patients with inadequate lymphadenectomies at non-academic centers, with survival significantly lower than in either lymphadenectomy group at academic institutions (all Holm-adjusted *p* < 0.001). Differences among the remaining groups were not statistically significant. In adjusted Cox models, inadequate lymphadenectomy was independently associated with higher mortality (HR 1.23, 95% CI 1.15–1.31), and treatment at non-academic facilities again conferred a higher mortality risk (HR 1.12, 95% CI 1.05–1.19). To evaluate whether the survival impact of lymphadenectomy adequacy varied by facility type, we tested an interaction between the two terms, which was not statistically significant.

When survival was stratified by COR and facility type, unadjusted KM curves showed the highest OS among patients receiving COR at academic centers and the lowest among those undergoing incomplete surgery at non-academic centers (Supplementary Fig. 4). Pairwise comparisons demonstrated differences across all groups (all adjusted *p* < 0.05). These findings were consisted with adjusted Cox models, in which incomplete oncologic surgery was associated with a substantially higher risk of death compared with COR (HR 1.38, 95% CI 1.30–1.48).

Across all Cox models, several other predictors showed consistent associations with mortality. Male sex, higher comorbidity burden, extrahepatic tumor location, and more advanced stage were each associated with increased mortality. Minimally invasive and robotic approaches were associated with lower mortality compared with open surgery. Chemotherapy omission, whether due to refusal, contraindication, or other reasons, was strongly associated with worse survival. Of note, the Cox proportional hazards assumption was violated in all cases. Sensitivity analyses censoring deaths within 90 days of surgery did not materially alter the survival patterns.

## Discussion

This national analysis of biliary tract cancers yielded three principal findings. First, academic programs deliver COR at higher rates than other facility types, and this translates into superior overall survival. This survival benefit was evident when assessing the individual components of COR – margin negativity and adequate lymphadenectomy – each of which independently predicted improved outcomes. Second, even in academic programs, fewer than half of cases met lymphadenectomy benchmarks. Third, among BTC subtypes, disparities were most pronounced for gallbladder cancer, for which both margin status and lymphadenectomy quality varied markedly by facility type. Collectively, these findings underscore the dual imperative to reassess lymph node staging protocols across centers and strengthen referral pathways to high-performing academic programs.

Previous studies have demonstrated that the type of treatment facility matters for individual BTC subtypes. Academic centers achieve higher negative margin rates, adequate lymphadenectomy rates, and improved survival rates in IHC [7, 8]. Another NCDB analysis found that, even after propensity-matching for resection type and adjuvant chemotherapy, patients at academic centers had lower mortality and improved overall survival [9]. Similarly, Dutch data showed higher resection rates and improved survival among patients with EHC managed at academic centers [17].

Across analyses, our assessment of BTCs as a unified cohort confirms and extends these observations, demonstrating higher rates of COR at academic programs. Although the absolute difference in negative margin rates between academic and non-academic centers was negligible (79.5% vs. 79.0%), margin status itself remained an independent predictor of survival in our Cox models, underscoring its continued relevance as a component of COR. Importantly, lymphadenectomy adequacy, the second component of COR, and facility type exerted independent and additive effects on survival, with both ≥ 6-node retrieval and academic centers conferring a survival advantage.

Despite the overall advantage of academic centers, adequate lymphadenectomy remains strikingly underperformed. Only 48.2% of academic cases met the ≥ 6-node threshold, consistent with prior registry reports [11, 18–20]. Several factors likely undermine consistent node retrieval. First, the biliary tract drains into multiple nodal basins [21], making dissections technically demanding, especially through a minimally invasive approach [11]. This challenge is most pronounced in GBC. Although our cohort excluded T1a tumors, which appropriately undergo simple cholecystectomy without lymphadenectomy, many patients with GBC still present after an initial non-oncologic operation. In such cases, completion radical cholecystectomy, necessary for both nodal evaluation and margin-negative resection, is performed in only a small proportion of patients [22]. In contrast, for IHC and EHC, neither margin negativity nor adequate lymphadenectomy differed significantly between academic and non-academic centers. This nuance suggests that facility-level interventions may need to be tailored, and that referral pathways and quality benchmarking may be most impactful for GBC.

Beyond these technical and subtype-specific barriers, guideline ambiguity further complicates practice. NCCN’s qualitative “site-appropriate” standard [4] lacks a numeric benchmark, which may lead surgeons to underestimate the required extent of dissection and to understage patients. Even in aggressive attempts, portal lymphadenectomies often yield as few as three or four nodes [23], despite evidence that examining six or more nodes provides meaningful therapeutic benefit in BTC [24, 25]. Still, some surgeons remain uncertain about the value of extensive lymphadenectomy, especially in clinically node-negative patients [19], leading to variability in practice across facility types. The magnitude of inadequate lymphadenectomy observed across centers in our data, and its association with a higher risk of death, may reflect this dissonance, highlighting the need for consensus on a standardized numeric benchmark to define adequacy.

Several factors likely underlie the advantage of academic centers. Multidisciplinary teams (MDTs) streamline coordination between surgery, radiology, and pathology while reinforcing adherence to guidelines [26–28]. In one institution, the implementation of a disease-specific MDT changed diagnoses in 38% of biliary cases and management in 35%, representing the highest impact among the cancers studied [28]. They attributed this to the resource-intensive nature of BTC management, which requires coordinated expertise across multiple specialties, including hepatopancreatobiliary surgeons, interventional radiologists, gastrointestinal body radiologists, and specialized pathologists.

Although facility type predicts outcomes more strongly than surgical volume in IHC, over half of academic centers are high-volume [8]. Our cohort revealed that 52.9% of resections at academic programs occurred at high-volume sites, compared to 15.0% at non-academic centers. These high-volume, academic programs benefit from concentrated referral streams that hone team expertise and promote best practices in the management of BTC [29–31]. Nevertheless, patient selection may also contribute to these observed advantages, as academic centers in our cohort more often operated on younger patients with fewer comorbidities who could travel farther for care.

### Limitations

The study should be interpreted in the context of multiple limitations. The NCDB lacks granular data on nodal station location, margin width, recurrence patterns, and postoperative complications. Although our ≥ 6-node cutoff is derived from the AJCC, access to nodal station data would have enabled evaluation of adequacy under other guideline standards. Still, our approach aligns with previous registry studies. Additionally, the study spans nearly two decades, during which operative standards, staging definitions, and lymphadenectomy practices have evolved, introducing temporal variability that cannot be fully accounted for in the NCDB. Because our analyses rely on Commission on Cancer facility designations, which are specific to the United States, the generalizability of our findings to international settings is limited. However, facility type was selected because it more accurately reflects the multidisciplinary resources and institutional infrastructure captured in the NCDB, which are central to the study’s aims. Finally, grouping IHC, EHC, and GBC under a single framework may obscure subtype-specific epidemiology, biology, and surgical complexity. However, our unified approach aligns with NCCN’s joint guidelines and our goal of identifying broad, system-level targets.

## Conclusion

Our findings suggest two complementary strategies to help address observed disparities in BTC surgery. First, strengthening referral pathways to academic centers, where coordinated expertise is associated with higher rates of complete oncologic resection and superior survival. Second, elevating surgical quality across all facilities through standardized nodal staging protocols is essential, given the persistently low rates of adequate lymphadenectomy even at academic programs. Since travel burdens can deter patients from accessing these high-performing programs [32, 33], health systems should consider expanding telehealth services for preoperative consultations, multidisciplinary case discussions, and follow-up care [34]. Embedding patient navigators within these networks may further mitigate geographic and logistical barriers by coordinating transportation and support services [35–37]. Ultimately, policies that support reimbursement of telehealth encounters and navigator services could play an important role in promoting equitable access to guideline-concordant care in BTCs.

## Supplementary Information

Below is the link to the electronic supplementary material.

Supplementary Material 1

Supplementary Material 2

Supplementary Material 3

Supplementary Material 4

Supplementary Material 5

## Data Availability

Data is available through the National Cancer Database.
