# Supplementary material for: Facility Type Predicts Completeness of Oncologic Resection and Survival in Biliary Tract Cancers
Source: J Gastrointest Cancer. 2026 Feb 19;57(1):47. doi: 10.1007/s12029-026-01421-1 (PMC12920415; doi:10.1007/s12029-026-01421-1)
Supplement: Supplementary file 1 — Supplementary Material 1 [file 12029_2026_1421_MOESM1_ESM.docx]

**Supplementary Table 1. Multivariable Model Assessing Odds of Complete Oncologic Resection.**

| **Outcome: Complete Oncologic Resection** | | **OR (95% CI)** |
| --- | --- | --- |
| **Age** |  | 0.99 (0.99-1.00) |
| **Sex** | **Female** | ref |
|  | **Male** | 1.06 (0.97-1.17) |
| **Race/Ethnicity** | **Non-Hispanic White** | ref |
|  | **Non-Hispanic Black** | 0.81 (0.71-0.93) |
|  | **Hispanic** | 0.82 (0.72-0.94) |
|  | **Asian** | 0.90 (0.77-1.06) |
|  | **Other** | 1.08 (0.88-1.33) |
| **Charlson Deyo Score** | **0** | ref |
|  | **1** | 0.97 (0.88-1.06) |
|  | **2** | 0.92 (0.79-1.08) |
|  | **3** | 0.94 (0.79-1.08) |
| **Primary Site** | **Gallbladder** | ref |
|  | **Intrahepatic bile duct** | 0.98 (0.86-1.12) |
|  | **Extrahepatic bile duct** | 5.03 (4.56-5.56) |
| **Stage** | **I** | ref |
|  | **II** | 1.31 (1.17-1.46) |
|  | **III** | 1.06 (0.94-1.19) |
| **Facility Type** | **Academic** | ref |
|  | **CCP** | 0.78 (0.61-1.00) |
|  | **Comprehensive CCP** | 0.82 (0.74-0.90) |
|  | **Integrated Network Cancer** | 0.94 (0.85-1.05) |
| **Surgical Approach** | **Open** | ref |
|  | **Minimally invasive** | 0.84 (0.76-0.93) |
|  | **Robotic** | 1.12 (0.93-1.34) |
