# Supplementary material for: Facility Type Predicts Completeness of Oncologic Resection and Survival in Biliary Tract Cancers
Source: J Gastrointest Cancer. 2026 Feb 19;57(1):47. doi: 10.1007/s12029-026-01421-1 (PMC12920415; doi:10.1007/s12029-026-01421-1)

**Supplementary Figure 2.** Stage distribution of biliary cancer resections by primary site. Bars represent the proportion of patients within each stage (I-III) for gallbladder cancer, intrahepatic cholangiocarcinoma, and extrahepatic cholangiocarcinoma.


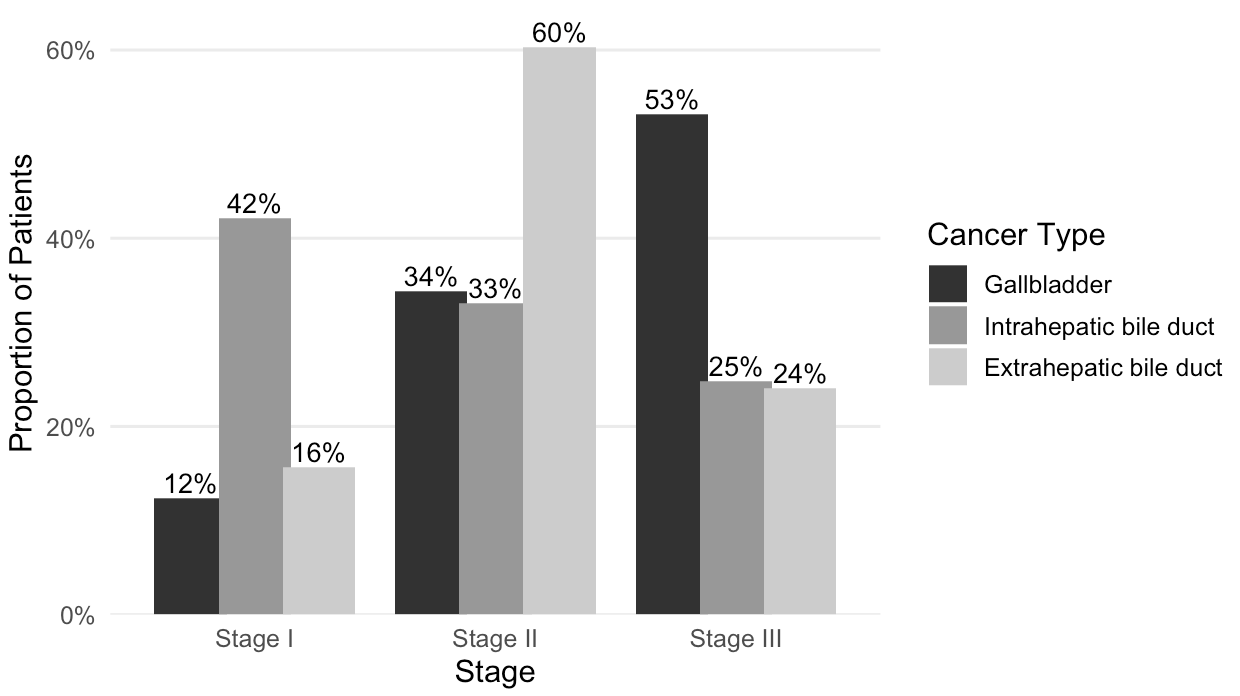

Supplement: Supplementary file 2 — Supplementary Material 2 [file 12029_2026_1421_MOESM2_ESM.docx]
