# Supplementary figures and images for: Facility Type Predicts Completeness of Oncologic Resection and Survival in Biliary Tract Cancers
Source: J Gastrointest Cancer. 2026 Feb 19;57(1):47. doi: 10.1007/s12029-026-01421-1 (PMC12920415; doi:10.1007/s12029-026-01421-1)

**Supplementary Figure 1. STROBE diagram of patient selection.**


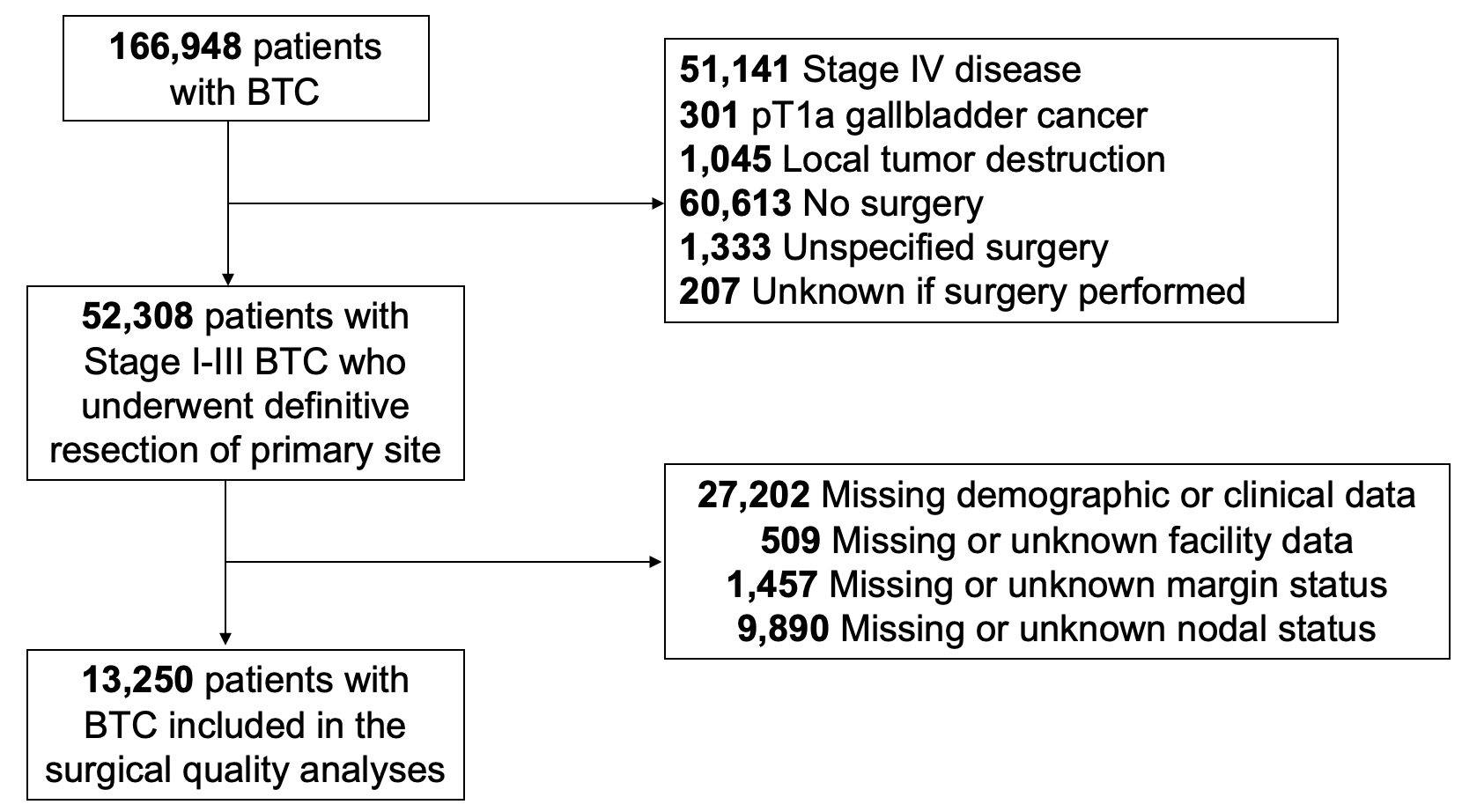

Supplement: Supplementary file 3 — Supplementary Material 3 [file 12029_2026_1421_MOESM3_ESM.docx]
