# Supplementary material for: Facility Type Predicts Completeness of Oncologic Resection and Survival in Biliary Tract Cancers
Source: J Gastrointest Cancer. 2026 Feb 19;57(1):47. doi: 10.1007/s12029-026-01421-1 (PMC12920415; doi:10.1007/s12029-026-01421-1)

**Supplementary Figure 4. Overall Survival by Complete Oncologic Resection, by Facility Type.** Kaplan-Meier curves for overall survival by complete oncologic resection by facility type (academic vs. non-academic). Risk tables indicate the number of patients at risk over time. Curves are truncated when the number at risk drops below 10% of the initial group size.


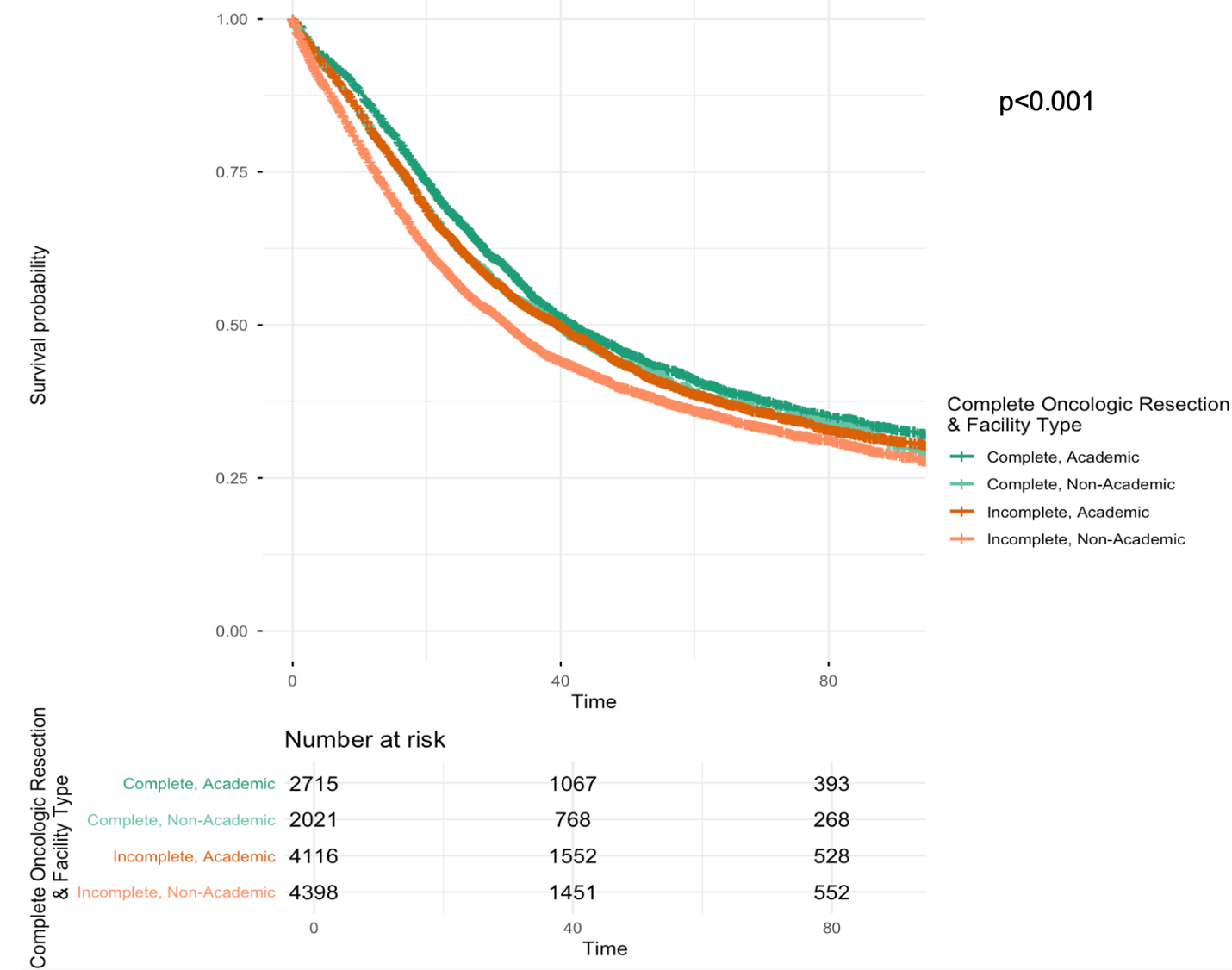

Supplement: Supplementary file 4 — Supplementary Material 4 [file 12029_2026_1421_MOESM4_ESM.docx]
